# Neurons in human motor thalamus encode reach kinematics and positional errors related to braking

**DOI:** 10.21203/rs.3.rs-6165736/v1

**Published:** 2025-03-26

**Authors:** Rex Tien, Jonathan Platt, Madelyn Mendlen, Drew Kern, Steven Ojemann, John Thompson, Daniel Kramer

**Affiliations:** University of Colorado Anschutz Medical Campus; University of Colorado Anschutz Medical Campus; University of Minnesota; University of Colorado Anschutz Medical Campus; University of Colorado Anschutz Medical Campus; University of Colorado Anschutz Medical Campus; University of Colorado Anschutz Medical Campus

## Abstract

Deep brain stimulation of the cerebellar-receiving region of motor thalamus, the ventral intermediate nucleus of the thalamus (VIM), effectively reduces the action tremor associated with essential tremor. However, the neural contribution of the VIM to the control of voluntary movement, and how that function relates to action tremor pathophysiology, is not well understood. In single thalamic neurons recorded during a naturalistic reaching task in essential tremor patients undergoing deep brain stimulation surgery, we find that firing rate changes align with the braking and stabilizing phases of reach movements, encode hand position and velocity above other kinematic variables, and strongly encode error signals relating the current hand position to the final reach target position. These findings support a hypothesis that the VIM contributes to the control of accurate stopping and stabilization of the hand, dysfunction of which aligns with models of action tremor generation.

## Introduction

Essential tremor (ET), the most common movement disorder^1^, is characterized by the presence of action tremor, defined as tremor occurring specifically during voluntary movement and postural maintenance^2^. The underlying cause of ET is associated with dysfunction of the cerebellum^3–5^, and neurosurgical interventions such as deep brain stimulation (DBS) that disrupt the cerebellothalamocortical pathway by targeting the ventral intermediate nucleus of the thalamus (VIM) have demonstrated strong efficacy in suppressing action tremor^6,7^. Despite the clinical evidence linking the VIM and action tremor, a mechanistic account of the functional role of the VIM in voluntary movement control, and how dysfunction in the VIM and its network leads to action tremor, remains unknown. Here, we investigate the relationship between VIM neuronal spiking activity and hand kinematics during a naturalistic reaching task and find support for the hypothesis that the VIM is involved in the control of braking and stabilization of goal-directed reaching movements.

The VIM receives input from the cerebellar deep nuclei (CDN)^8,9^ and projects to motor and premotor cortices^10,11^. Animal studies suggest that the CDN convey information about ongoing goal-directed movements to influence cortical motor output via the thalamus. Studies in monkeys have found that Purkinje cells, which project to the CDN, encode information relevant to reaching movements including the target position^12^, reach distance and direction^13^, and other kinematic features^14^. Monkey dentate nucleus neurons activate during visually guided reaches, with modulation time-aligned to specific events in learned reach sequences^15^. Cooling of the CDN results in a tremor-like oscillation after arm perturbation^16^. In mice, ablation of interpositus nucleus neurons results in over-reaching and inaccurate reaches^17^. Neurons in the mouse interpositus nucleus fire maximally around the time of reach braking and display speed-dependent firing rates (FRs), and optogenetic activation and deactivation of these neurons causes early and late braking, respectively^18^. These findings strongly suggest that CDN output assists in online movement correction for accurately acquiring and stopping at goal-directed reach targets. Human neuroimaging studies support these animal results, identifying increased activation in the cerebellum during visually-guided reaching^19,20^ and acquisition of targets requiring greater precision^21^.

Electrophysiological studies of cerebellar-receiving motor thalamus in monkeys provide evidence of neural activity related to target-directed movements. In monkeys, the thalamic ventral posterolateral nucleus pars oralis (VPLo) and Area X are likely homologous to human VIM^22,23^, receiving input from the CDN^9,24^. Neurons in these thalamic areas encode reach direction^25,26^ and duration of velocity^27^, with modulation specific to visually guided movements^28^ and displaying variable timing relative to movement onset^29^. Inactivation of VPLo led to hemiplegia, with deficits manifesting specifically in later reach stages as the hand approached the target^30^.

Electrophysiological studies in human VIM evince neuronal spiking synchronous with tremor^31–34^ and broadly responsive to passive and/or active movements of the contralateral limbs and face^33,35–38^. A few recent studies in humans have utilized structured behavioral tasks to show that VIM neuron activity relates to parameters of voluntary movements. Patil et al (2004) found that 81% of purported VIM neurons were modulated with grip force, and that grip force was decodable from small ensembles of VIM neurons^39^. Hanson et al (2012) recorded ensembles of VIM neurons while patients performed a 1D cursor target tracking task using a haptic glove^40^. They found that neurons were tuned to combinations of task features, including visual target appearance, movement, target direction and tremor.

Together, these studies in animals and humans suggest that the CDN and VIM play central roles in the control of goal-directed voluntary movements, specifically for accurately acquiring movement targets^12–17–21–26–28–30^ and maintaining active postures^16,31–34^. We hypothesize that the VIM acts as part of a cerebellothalamocortical circuit which monitors and controls reaching movements to accurately brake and stabilize the hand at reach targets. Within this framework, it would follow to predict that VIM neurons fire preferentially during deceleration of the hand, with activity encoding the direction of movement as well as information about the location of the hand relative to the final reach target. To test these predictions, we leveraged the unique setting of awake DBS implantation surgery to record the activity of VIM spiking units while subjects performed a naturalistic center-out-and-back reaching task. The 3D position of the hand was tracked using a novel application of deep learning based markerless motion tracking techniques, allowing for precise alignment of neural activity to the temporal phases and kinematics of reach movements^41^. We find that VIM spiking unit FRs modulate in the peri-movement period, with timing that is consistent with a role in movement braking, and to a lesser extent, stabilization. Furthermore, we find that many VIM units are differentially active for reaches in different directions, most prominently during the braking and stabilization phases. Regression analyses reveal that VIM unit FRs are most strongly related to hand position, error (position relative to target) and velocity terms. These findings support the hypothesis that the VIM is involved in the control of reach braking and stabilization at reach targets.

## Results

**Figures 1:**
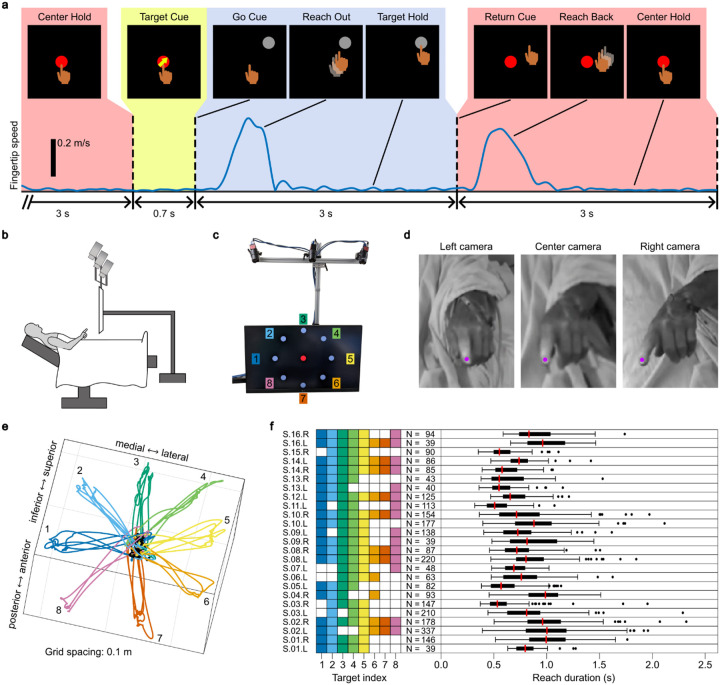
Intraoperative experimental design and kinematic extraction. **a**, Sequence and timing of the center-out-and-back task events. Subjects were instructed to maintain a pointing position over the center target (Center Hold). The disappearance of the center target and appearance of the outer target served as the Go Cue to reach to and hold at the new target. After a Target Hold period, the subject was instructed to reach back to the center target (Return Cue). This out-and-back sequence was then repeated with a new outer target. **b**, Schematic of the experimental setup in the operating room. The task display monitor and three overhead cameras were positioned above and just out of reach of the subject. **c**, The task display monitor, with all potential outer targets labeled. During the task, targets were displayed sequentially one at a time. **d**, A single, cropped frame from each camera view of subject S.14 performing the task. Magenta dots indicate the estimated position of the fingertip in each view as output by DeepLabCut. **e**, 3D fingertip position data from an entire task session (Procedure S.14.R, Session 2). Colors and number labels indicate the active outer target for each out-and-back segment, corresponding to the labels in c. Black trajectories indicate Center Hold periods. **f**, Summary of reach behavior for all subjects. “S.14.R” denotes Subject 14, right hemisphere procedure; “L” denotes left hemisphere. Colored squares indicate the targets that were visible to the subject and utilized during each procedure, i.e. not visually obstructed by operating room equipment. “N” denotes the number of valid reaches performed in each procedure. Box-and-whisker plots display the distribution of reach durations in each procedure; red lines indicate medians, boxes indicate the interquartile range, whisker length is 1.5 times the interquartile range, black markers represent outliers.

Data were obtained from 25 single-hemisphere procedures in 16 subjects (5 female) aged 70.2 ± 6.9 years (mean ± standard deviation) undergoing VIM DBS surgery for the treatment of ET. During the microelectrode recording phase of DBS surgery, subjects performed the center-out-and-back reaching task (Fig. 1a–c) for a total of 77 sessions across all subjects (3.08 ± 1.52 sessions per procedure). Of these 77 sessions, 7 were excluded due to a lack of identifiable spiking activity in the corresponding neural recordings, and 3 were excluded due to an inadequate number of successful reaches. After kinematic extraction (Fig. 1d,e), 44.15 ± 5.60 reaching movements (outward and returning) were isolated per session, for a total of 2,958 reaches. Of these collected reaches, 85 (2.9%) outliers were excluded for duration (greater than 1.5 times the interquartile range above the median for each procedure), leaving a total of 2,873 reaches for analysis (Fig. 1f). The average reach duration, defined as the time between Reach Start and Reach End (see Methods) was 799 ± 254 ms.

**Figures 2:**
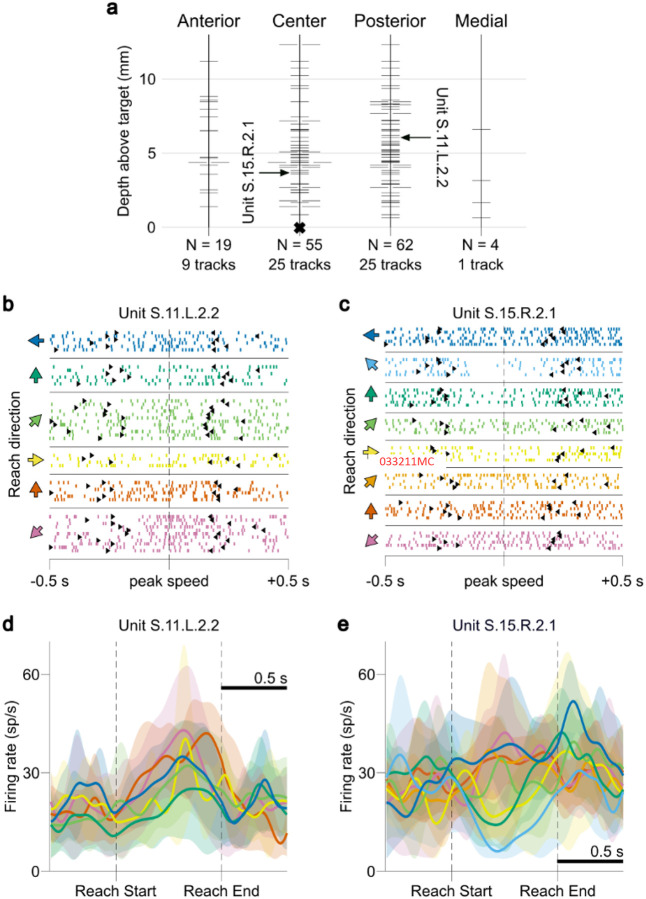
Neural data acquisition and examples of ventral intermediate nucleus unit spiking activity. **a**, Recording locations of all sorted spiking units across all subjects and sessions. “Center” indicates the electrode trajectory track aligned with the imaging-based surgical target. Anatomical direction labels indicate locations of secondary or tertiary recording electrodes in 2 mm offsets using a Ben-Gun arrangement. Multiple ticks at a single depth indicate that multiple units were sorted on a single electrode during a session. Depth values are relative to the target insertion depth derived from pre-surgical imaging. Arrows mark the recording locations of the units with activity depicted in **b-e. b**, Raster plot of a spiking unit from procedure S.11.L session 2. Each row displays the spike times during a single reach movement, aligned at peak fingertip speed. Black arrows indicate Reach Start and Reach End times. Colors indicate the cued direction of each reach, with colored arrows indicating the direction of reach from the subject’s perspective. **c**, Same as in **b**, but for a spiking unit from procedure S.15.R session 2. **d**, Firing rate of the unit shown in **b**, averaged across all reaches in each direction; colors are the same as in **b**. Shaded regions depict 1 standard deviation above and below the mean. **e**, Same as in **f**, but for the unit depicted in c. Firing rate changes clustered around the middle and end of the reach, and directional-dependent differences in firing can be observed.

For each session, neural recordings from all microelectrodes were sorted offline to identify single- and multi-unit spiking activity, resulting in a total of 138 isolated units (0.78 ± 0.62 per microelectrode recording). The mean FR over all analyzed units was 19.82 ± 15.03 spikes/s. Microelectrode recording depths ranged from 0.65 to 12.32 mm above the radiographically determined clinical DBS target (Fig. 2a).

### Ventral intermediate nucleus unit firing rate modulation occurs predominantly during late reach

**Figure 3:**
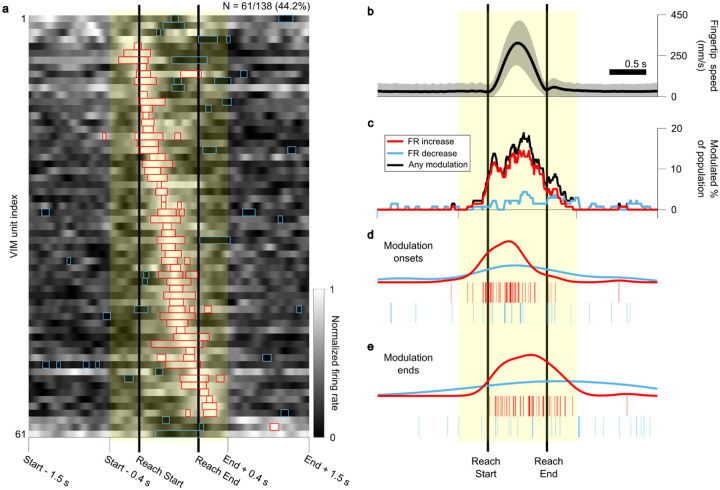
Ventral intermediate nucleus unit firing rates are modulated during reach movements. **a**, Mean firing rates (FRs) of units with significant modulation in the peri-reach time window (yellow shaded region; 400 ms before Reach Start to 400 ms after Reach End), averaged across all reaches in a session and normalized from 0 (black) to 1 (white) within the plotted time window. Units are sorted according to the time of peak mean FR. Red boxes indicate periods of significant positive FR modulation; blue boxes indicate periods of significant negative FR modulation above or below a shuffle-derived baseline distribution of mean FRs, (10,000 shuffles, p < 0.01, two-sided, for at least 5 consecutive samples [50 ms on average]). **b**, Peri-reach fingertip speed profile averaged across all reaches in all sessions. **c**, Proportion of total recorded units displaying significant modulation at each timepoint. The red and blue lines denote the proportion of units with significant positive or negative modulation, respectively. The black line denotes the proportion of units displaying any significant modulation. **d**, Onset times of significant modulation periods. The red and blue ticks denote the start times of periods of significant positive and negative modulation, respectively. The red and blue curves are kernel density estimates describing the density of red and blue ticks, respectively. **e**, End times of significant modulation periods. Colors are the same as in **d**. Periods of significant modulation generally start after reach has begun and end in late reach periods. The yellow shaded region in **b-e** denotes the peri-reach time window.

VIM unit FRs were calculated at 10 ms average increments aligned to both Reach Starts and Reach Ends (see Methods) and averaged across all reaches per session (regardless of reach direction) to determine if unit FRs were modulated in concordance with specific temporal reach phases. Significant FR modulation was observed in 44.2% (61/138) of units (mean FR greater or less than 99.5% of shuffle derived baseline samples, 10,000 shuffles, p < 0.01, two-sided, for at least 5 contiguous samples) in the peri-reach window from 400 ms before Reach Start to 400 ms after Reach End (Fig. 3a). Most peri-reach FR modulation was positive, with 79.1% (1400/1770) of the significantly modulated peri-reach samples corresponded to FR increases.

Significant modulation periods predominantly occurred after Reach Start (Fig. 3c–e). Pre-movement modulation was present but modest; 9.4% (13/138) of units were significantly modulated before Reach Start, however only 5.7% (101/1,770) of all significant peri-reach modulated samples occurred in this pre-movement period. FR modulation was significantly more likely to occur in the period between Reach Start and Reach End compared to periods before Reach Start and after Reach End (Chi-square test, p < 0.01), with 79.0% of significant samples occurring during the reach. Modulation periods were found to be significantly biased toward the latter half of reach when the hand was decelerating, with 53.0% occurring in the second half compared to 47.0% in the first half (Chi-square test, p = 0.026). The peak number of simultaneously modulated units in the entire peri-reach window (26/138 units, 18.8%) occurred during the late reach period, at 490 ms after Reach Start on average (61.3% of the time between Reach Start and Reach End, Fig. 3c). Moreover, modulation onsets were most likely to occur during early reach, and modulation ends occurred mostly during late reach (Fig. 3d,e). Modulation subsequently decreased in the stabilization and target acquisition period (0–400 ms after Reach End), with 14.5% (20/138) of units exhibiting any modulation after Reach End. This stabilization period contained 15.3% of all modulated peri-reach samples, which was significantly greater than the proportion observed before Reach Start (Chi-square test, p < 0.01).

The timing of peri-reach FR modulation was preserved when analyses were performed with real-time sampling, instead of the time-stretching technique described above (Extended Data Fig. 1). FR modulation was not observed to coincide with the delivery of the Target Cue (Extended Data Fig. 2a) or the Go Cue (Extended Data Fig. 2b). Aligning neural activity to the time of peak fingertip speed in each reach confirmed the observation that FR modulation was most prevalent during reaches, and was biased toward the deceleration phase, after peak speed (Extended Data Fig. 2c).

### Ventral intermediate nucleus units are tuned to reach direction

**Figure 4:**
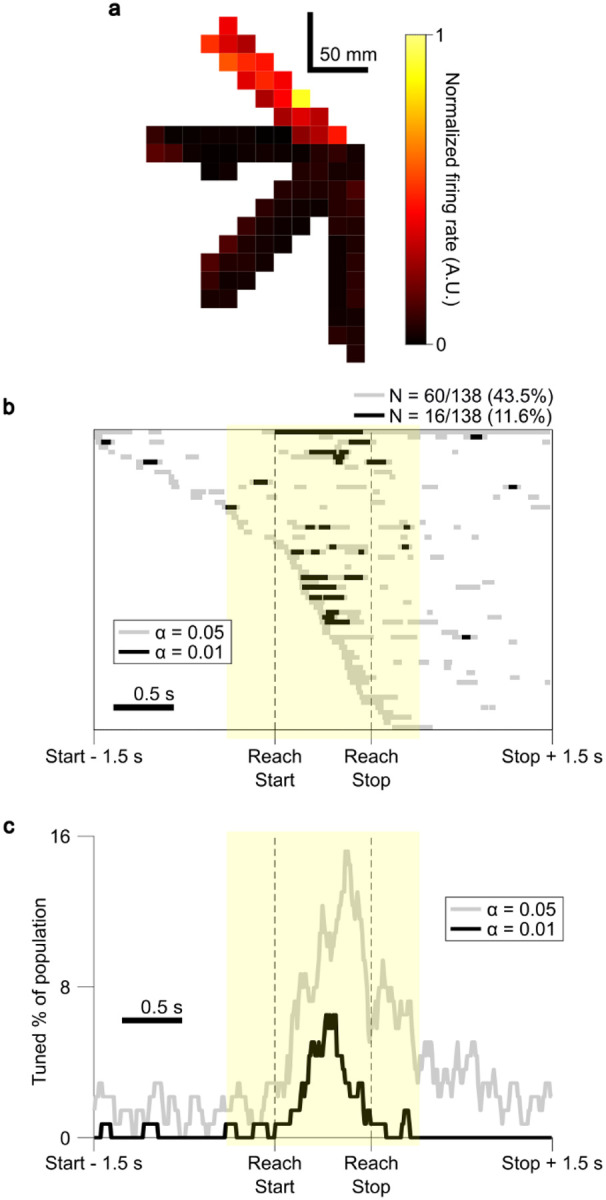
Ventral intermediate nucleus unit firing rates vary with reach direction. **a**, Spatial activation heatmap of a unit from Procedure S.02.L Session 3. Four outer targets were used in this session. Colors indicate the normalized mean FR observed while the fingertip was in each 15-by-15 mm square. For visualization, 3D fingertip positions were projected into the top two principal components and rotated to align approximately with the actual target positions as presented on the task monitor. Regions with no heatmap entry were never traversed by the fingertip. **b**, Timing of significant directional tuning. Each row represents a significantly tuned unit, and boxes demarcate periods of significant tuning (Kruskal-Wallis ANOVA across reach directions, p < 0.05 [gray] or p < 0.01 [black] for at least 5 consecutive samples [50 ms] in the peri-reach time window [yellow shading]). **c**, The proportion of total units that were significantly tuned to reach direction at each timepoint (colors are the same as in **b**). Directional tuning was biased to occur during later reach phases.

The center-out-and-back reaching task was designed to elicit movements in a range of directions. In each session, subjects reached to 5.53 ± 1.86 outer targets (Fig. 1f); not all targets were visible to all subjects due to visual obstruction by the stereotactic headframe or other operating room equipment. This variation in reach direction allowed for further analysis of VIM unit FRs for evidence of systematic relationships to reach kinematics. Initial visualization of mean FRs across the movement space revealed structured spatial variation in FRs during the task (Fig. 4a).

At each peri-reach timepoint, FRs were subjected to a one-way Kruskal-Wallis ANOVA across reach directions (see Methods). Significance for directional tuning was assessed by whether the unit exhibited Kruskal-Wallis ANOVA significance at an α-level of 0.05 for at least 5 contiguous samples (50 ms) in the peri-reach time window. We did not test whether units adhered to a smooth directional tuning function such as cosine tuning^42^ but simply required that units exhibit significantly different FRs for at least one reach direction compared to others. Based on these criteria, 43.5% (60/138) of units were determined to be significantly directionally tuned (Fig. 4b). The timing of these periods of significant directional tuning was similar to the pattern observed for overall peri-reach modulation (Fig. 4c): 67.7% of significant directional tuning samples fell between Reach Start and Reach End, and samples were biased to occur in the second half of the reach period (63.8% in the second half vs. 36.2% in the first half, Chi-square test, p < 0.01). Significant directional tuning samples were also more likely to occur during the reach than before Reach Start or after Reach End and were more likely to occur after Reach End than before Reach Start (Chi-square tests, p < 0.01).

### Ventral intermediate nucleus unit firing rates encode reach kinematics

**Figure 5:**
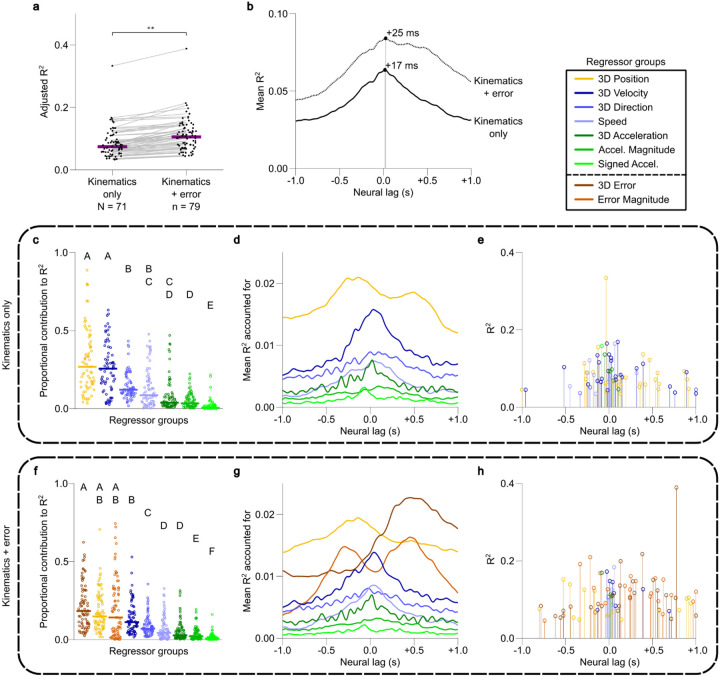
Ventral intermediate nucleus unit firing rates encode kinematics and error terms at a range of lags. **a**, Adjusted R-square values for units with significant regression fits (observed R-square greater than random expected R-square, firing rate [FR] sample times randomly shifted 10,000 times, p < 0.01) for FRs regressed against fingertip kinematics only or kinematics and error terms (error defined as the difference between instantaneous and goal fingertip position). Horizontal bars indicate medians and gray lines connect adjusted R-squares for units with significant fits for both models. Adjusted R-square was obtained at the optimal lag for each unit and model, in the range −1 to +1 s (FRs time-shifted relative to kinematics). Medians compared with Wilcoxon Rank Sum Test (p < 0.01). **b**, Mean R-squares across all units with significant regression fits for the two models across lags. Markers denote the lags at which peak mean R-squares were observed. **c**, Proportional contribution of each kinematic regressor group to total R-square, as determined by Shapley decomposition at each unit’s optimal lag. Each unit with a significant regression fit has one entry in every column. Horizontal bars denote medians, and capital letters denote significance groups (groups with the same letter label had medians that were not statistically different; Wilcoxon Rank Sum Test, α = 0.01). **d**, Mean R-square accounted for by each regressor group across lags, determined by Shapley decomposition. **e**, Maximum R-square and associated optimal lag for each unit with significant regression fit to kinematics, colored according to the kinematic regressor group which contributed the most to the unit’s R-square, determined by Shapley decomposition. **f, g, h**, Same as in **c, d, e**, but for regressions of FRs against kinematic and error terms. Position and error terms were the most strongly predictive of FRs at a range of optimal lags.

To determine whether FRs encoded instantaneous reach kinematics, encoding models were built using multiple linear regression. FRs were regressed against a comprehensive set of continuous fingertip kinematic features including position, velocity, speed, acceleration, acceleration magnitude, and signed acceleration, taking data from time windows 250 ms before Reach Starts to 250 ms after Reach Ends. A range of neural-to-kinematic lags were tested by offsetting the FR sample times in 8.3 ms steps from – 1,000 ms to + 1,000 ms relative to the kinematic data. Significant kinematic encoding was observed in 51.5% (71/138) of units (observed R-square greater than 99% of random expected R-squares generated by randomly shifting FR indices against kinematic indices 10,000 times [p < 0.01, one-sided]). Significant kinematic regressions resulted in a mean R-square of 0.086 ± 0.046 as calculated at each unit’s optimal lag. The overall optimal lag for kinematic encoding (the single lag at which the maximum mean R-square across all units was observed) was + 17 ms (neural data lagging kinematic data, Fig. 5b).

Shapley decomposition was employed to calculate the contribution of each kinematic term to overall model R-squares (see Methods). 3D position and 3D velocity were found to be the top proportional contributors to R-square (Fig. 5c) when evaluated at each unit’s optimal lag (Wilcoxon Rank Sum Test, p < 0.01). While R-square contributions from velocity terms were clustered around zero lag, positional contributions were more distributed, with peaks at both negative and positive lags (Fig. 5d). Of the statistically significant kinematic encoding units, 38.0% (27/71) had 3D position as the top explanatory regressor group, and 35.2% (25/71) had 3D velocity as the top explanatory regressor group (Fig. 5e).

### Ventral intermediate nucleus unit firing rates encode error terms

To investigate whether VIM units encoded information about the final reach target relevant to reach movement control, regressions were repeated with 3D error and error magnitude terms included in an augmented set of regressors, along with the full set of kinematic regressors. Here, 3D error is defined as the vector difference between the instantaneous fingertip position during the reach and the final average hold position of the fingertip after the reach. Error magnitude is defined as the magnitude of the 3D error vector (the Euclidean distance between the instantaneous fingertip position and the mean final hold position). With these error terms included, 57.3% (79/138) of units were found to significantly encode fingertip kinematics and error (regression fit significance calculated as with “kinematics only” regressions above, α = 0.01). The inclusion of error terms resulted in a significant increase in median adjusted R-square when each unit was evaluated at its optimal lag for each model (Fig. 5a, Wilcoxon Rank Sum Test, p < 0.01). For the 59 units which displayed significant fits to both the “kinematics only” and the “kinematics + error” models, the average adjusted R-square increase was 0.026. In models that included error terms, the peak average R-square over all significant units was observed at a lag of + 25 ms (Fig. 5b).

Utilizing Shapley decomposition at each unit’s optimal lag revealed that 3D error and error magnitude terms were among the top proportional contributors to R-square values along with 3D position, with the median proportional contribution from 3D error significantly exceeding the median proportional contribution from 3D velocity (Fig. 5f). Error terms were especially predictive of FRs at non-zero lags, with error magnitude contributing at both negative and positive lags, and 3D error more informative at positive lags (Fig. 5g). Error terms were the largest single-group contributors to R-square for 62.0% (49/79) of units with significant regression fits, with 3D error and error magnitude as the top contributor for 32.9% (26/79) and 29.1% (23/79) of these units, respectively (Fig. 5h). These results indicate that error information relating the current fingertip position to the goal position was encoded in VIM unit FRs above and beyond the encoding of kinematics, especially at non-zero lags.

To examine the timing of kinematic and error encoding, zero-lag regressions were performed on data from 500 ms windows centered on either Reach Starts or Reach Ends. A significantly larger proportion of units displayed kinematic and error encoding at Reach Ends than at Reach Starts (two-tailed equality of proportions t-test, p < 0.01), with 29.7% (41/138) of units found to have significant regression fits when regressed against kinematic and error data from Reach End time windows (significance calculated as for other regressions above, p < 0.05), as compared to 11.6% (16/138) of units for Reach Start time window data (Extended Data Fig. 3). This implies that target-oriented movement encoding is especially prevalent in VIM around the time of stopping and target acquisition, as compared to movement initiation.

## Discussion

We report movement-responsive spiking neural activity in the VIM during an intraoperative, naturalistic center-out reaching task. Aligning VIM unit activity to 3D reach kinematic events revealed that changes in neural activity were significantly biased to occur in the latter half of reach, with timing suggestive of a role in the control of braking, stopping, and stabilization. Modulation was observed most consistently from just before deceleration to just after movement end (Fig. 3). Additionally, neural modulation was significantly more likely in the post-reach period compared to pre-reach. Directional tuning was also observed to occur primarily in the same braking and stabilizing time windows (Fig. 4c). We observed an increase in the predictive relationship between kinematic terms and neural activity when including a measure of “error” from the intended target (Fig. 5a), and more units significantly encoded kinematic and error terms around the time of Reach End compared to Reach Start (Extended Data Fig. 3), suggesting a higher-order, target-oriented function for the VIM in the control of braking and stabilization. Overall, these findings support a role for the VIM in successfully and accurately stopping goal-directed reach movements.

In support of a brake-related function for cerebellar-receiving thalamus, temporal correspondence between neural activity and the later stages of reach movements has been observed in monkey ventrolateral thalamus^26^ and mouse DCN^18^. In the latter, optogenetic excitation of the DCN stopped reaches early. Furthermore, inactivation of monkey ventrolateral thalamus results in deficits specific to the time around reach target acquisition^30^. However, in contrast, several animal studies present evidence suggesting that cerebellar-receiving thalamus is critical for movement initiation, identifying pre-movement neuronal activation in mouse cerebellar-receiving thalamus^43^ and monkey ventrolateral thalamus^25,29^. These three studies focused solely on pre-movement activation and did not investigate later movement periods; deceleration-related activation patterns may have been observed if those time periods were assessed. We did observe that 9.4% of VIM units were modulated before movement started, implying that the VIM may have some involvement in movement initiation. However, much greater neural engagement was observed after movement initiation in our data (94.3% of all significantly modulated peri-reach timepoints), with modulation biased toward the deceleration and target acquisition stages of reach, implying that the primary role of the VIM is in the control of goal-directed movement braking.

VIM units were also responsive to variations in reach direction and continuous movement kinematics. A large portion of units displayed significantly different FRs for movements in different directions (60/138, 43.5%, α = 0.05), particularly around the time of reach braking and target acquisition (Fig. 4b,c). To identify which specific aspects of movement explained neural variability, VIM unit FRs were regressed against a broad set of continuous kinematic features at a range of lags. This analysis revealed that the majority of units significantly encoded kinematics (71/138 units, 51.5%, α = 0.01), with hand position and velocity being the features most strongly encoded in VIM unit FRs. Directional sensitivity has been identified previously using highly constrained tasks in human VIM^40^, monkey ventrolateral thalamus^25,26^ and cerebellum^13,14^. The directional sensitivity and kinematic encoding present in VIM units together suggest that VIM is involved not merely in the general timing of movement braking, but also in controlling specific movements to brake accurately at targets when approaching from different directions.

Individual VIM units displayed a range of optimal kinematic encoding lags (the temporal offset between FRs and kinematics that resulted in the highest mean regression R-square). Both negative and positive lags were observed, and lags were clustered near zero (Fig. 5). For pure kinematic encoding, the overall optimal lag was +17 ms. This short positive optimal lag suggests that, despite its projections to motor regions of cortex^10,11^, VIM is not a purely “motor” structure, as negative optimal lags in the range of −50 ms to −150 ms are typically observed in primary motor cortex^44^, and a +17 ms lag would be inappropriate for driving movement in a feedforward manner. However, the presence of negative optimal lags in individual units, as well as the proximity of the average optimal lag to zero suggest that VIM unit activity reflects a combination of motor commands and feedback information. Similar distributions of optimal kinematic encoding lags have been observed in primary somatosensory cortex^45^, which receives information about voluntary movement in addition to peripheral somatosensory feedback afference^46^. However, the coincident timing of VIM unit modulation with reach braking and the distribution of optimal kinematic encoding lags imply that the VIM is not a purely “sensory” reactive positional encoder or proprioceptive relay station. If the VIM were a reactive kinematic encoder, we would expect to observe consistent positive optimal lags and a more even distribution of activity modulation across movement phases.

Given the observed characteristics of VIM neural activity, and that the VIM connects the cerebellum and the motor/premotor cortices (with strong bidirectional communication^11^), we hypothesize that the VIM functions as a component of an optimal forward model feedback controller for reach braking and stabilization. Modeling the cerebellothalamocortical circuit as an optimal feedback controller that utilizes a forward model has been an increasingly popular and useful approach in the study of motor control^9,21,47,48^, with support from neurophysiological evidence in the cerebellum^49–52^ and ventrolateral thalamus^53,54^. This model predicts that a component of the controller would encode the difference between the goal position and estimated current hand position as an “error” signal. To evaluate the possibility that the VIM plays this role, we performed additional regressions, adding positional error and error magnitude terms to the set of kinematic regressors. We found that error terms were strong predictors of variability in VIM unit FRs, resulting in significant increases in model fit over models that featured only kinematic terms (Fig. 5a). Directional error and error magnitude terms were the most strongly encoded regressors for 62.0% (49/79) of the units with significant regression fits. This was especially true for units with non-zero optimal lags (Fig. 5h).

Engagement of a dysfunctional cerebellothalamic braking and stabilizing control system could explain the observation that ET is characterized by an action tremor. Action tremor, by definition, occurs with goal-directed movements or postural holding, which is primarily where engagement of a braking/stabilizing system would occur. Dysfunction specific to braking and stabilizing would explain the typical absence of tremor in contexts of rest (minimal engagement), and worsening tremor with high precision actions such as drinking or writing^55^ (maximal engagement). Even the rest tremor observed in late-stage ET can be explained by this model if rest is considered a low-level postural maintenance activity and as ET progresses, even this low engagement is affected. Modeling the VIM as part of a forward model feedback controller for movement braking and stabilization fits into this schema and recent modeling studies support this view, where dysfunction in a forward model controller for maintaining position (in the form of delays or prediction errors) leads to cyclical overcorrections and a resulting tremor^56,57^. This provides a link between VIM circuit dysfunction and the action tremor of ET. Furthermore, if the VIM processes and transmits error information related to successful movement braking and stabilizing, and this information is dysfunctional in ET, removing the dysfunctional error information would prevent the cyclic overcorrection that leads to tremor. This is one possible explanation for the success of targeting the VIM for tremor while having a limited effect on the other aspects of movement such as initiation (although notably ataxia is one side effect of VIM DBS^58^, which is a predicted outcome of disrupting the error signal in a forward model controller for braking). In total, this study presents evidence that the VIM plays a role in braking and stabilizing and may act as a processor of error signals.

Our findings should be considered in the context of several limitations. First, the posture of the subjects during surgery and the use of a stereotactic headframe meant that subjects were not always able to see all 8 potential targets. In such cases, the subset of visible targets was used. We utilized a relatively short duration requirement for detection of significant FR modulation and directional tuning (50 ms), though results were not overly sensitive to the length of the required minimum duration of significance (Extended Data Fig. 4). Additionally, subjects received no on-screen feedback about their pointing accuracy, making online correction difficult and post-hoc estimation of error only approximate. In terms of localization of recorded units, while electrode trajectories were based on initial MRI imaging, and trajectories were confirmed post-hoc using intraoperative CT and atlas-based methods, we cannot say for certain that the units we recorded were always located in anatomical VIM or the exact cerebellar receiving area of thalamus, and some units were likely located in adjacent nuclei including the ventralis oralis anterior and posterior (Voa/Vop)^23^. Furthermore, there was some selection bias in which units were recorded during experimental sessions, as units with high baseline FRs that were generally responsive to voluntary movement (as determined by the clinical team) were studied preferentially. Nevertheless, no systematic relationships were observed between either the depth of recording or electrode track location, and the likelihood of observing significant peri-reach FR modulation, directional tuning, or kinematic encoding (Extended Data Fig. 5). Our task involved moving between periods of active postural maintenance, and thus direct comparison of our results to studies which involved movement from rest are difficult. This work attempted to understand the function of the VIM, but in ET patients who inherently have motor system dysfunction, limiting the generalizability of our conclusions without further study in healthy subjects or animal models. Our analyses did not focus specifically on identifying tremor cells in the VIM or on hold periods where tremor was prominent. Finally, ET is a heterogeneous disease with a variety of clinical features^55^ and modeling the VIM as part of a forward model controller may be limited to explaining action tremor and derivatives like intention tremor.

In this work, we utilized novel intraoperative motion tracking methods and a standard, unconstrained naturalistic movement task to study VIM neuronal activity alignment with movement phases and encoding of kinematics. This method allowed us to identify that VIM unit FRs modulated in time with movement braking, as opposed to movement initiation or postural holding. VIM units were also found to encode movement direction, as well as positional and error terms at various lags, and velocity at a short lag. We posit that these results provide evidence that VIM acts as part of a cerebellothalamocortical circuit for accurate control of movement braking and stabilization. This provides a possible explanation for how the VIM, ET, and action tremor relate, and suggests a framework for better understanding the neural basis of accurate movement control.

## Methods

### Subjects

This study was approved by the Colorado Multiple Institution Review Board (COMIRB #20–2979). Subjects were recruited through the University of Colorado Anschutz Medical Campus Movement Disorders Center. All participants were diagnosed with ET according to the standards of the Movement Disorder Society, received standard-of-care clinical evaluation for ET-DBS candidacy, and elected to undergo DBS surgery before being contacted by our research team. Written informed consent was obtained from all subjects prior to surgery.

### Behavioral task

Task instructions were displayed on a 43 in computer monitor attached to a custom-built mobile cart via an industrial monitor arm (Inverted Elite 5220 Double Arm LCD Monitor Mount, ICWUSA.com, LLC, Medford, OR) used to position the monitor above and just out of reach of the subject (Fig. 1b). The monitor position was adjusted to maximize the number of targets visible to the subject, and the cart position was marked so that all subsequent behavioral sessions were performed with the monitor in the same location relative to the subject.

All behavioral sessions occurred during the awake neurological testing and microelectrode recording phase of DBS surgery. After completion of the clinical neurological examination at each microelectrode stopping point, if at least one distinguishable spiking unit was deemed to be present by the experimenters, the task display monitor was brought into place over the subject and the behavioral task commenced.

The behavioral task was a “center-out-and-back” reaching paradigm, consisting of a central target and 8 outer targets evenly spaced around a circle of radius 178 mm from the central target. Target circles were 30 mm in diameter. Subjects were instructed to point at and hold position over the currently visible target with their index finger, and when the displayed target changed, to point at the new target as quickly and accurately as they were able. The behavioral task was always performed with the hand contralateral to the recorded brain hemisphere. The task was displayed using MATLAB (The MathWorks, Inc., Natick, MA) and the open-source package Psychtoolbox-3 (http://psychtoolbox.org). Task event display timestamps were saved for further analysis and a synchronizing voltage pulse was sent from the task display computer to the neural data acquisition system at the start of each trial for data alignment.

The task proceeded as follows: the central target was displayed for 3 s, followed by a Target Cue phase wherein an arrow was overlayed on the center target for 0.7 s indicating the direction of the upcoming outer target. The central target and cue were then removed, and an outer target was displayed for 3 s. The outer target was then removed and the central target shown, and the next trial began (Fig. 1a). Outer targets were displayed in a pseudorandom, load-balanced fashion, cycling through all visible outer targets until at least 24 out-and-back sequences (48 individual reaches) were presented.

### Kinematic data acquisition and preprocessing

Subjects’ hand and arm movements were recorded using three 1.6 megapixel monochrome Blackfly USB3 video cameras (Teledyne FLIR LLC, Wilsonville, OR) mounted above the task display monitor. Cameras were synchronized and controlled using custom Python software, and captured video at a rate of 120 frames/s. The cameras produced a voltage pulse upon each frame capture which was sent to the neural data acquisition system for data alignment.

The position of the distal tip of the index finger was reconstructed using the open-source packages DeepLabCut^59^ (https://github.com/DeepLabCut/DeepLabCut) and Anipose^60^ (https://anipose.readthedocs.io). DeepLabCut is a deep learning based computer vision package that utilizes a pretrained deep convolutional neural network to estimate the location of keypoints of interest in video frames after training using manually labeled input frames. DeepLabCut was used to estimate fingertip position based on 50 manually labeled frames per camera per recording session, using default training parameters. Only frames with fingertip localization likelihood score greater than 0.95 as output by DeepLabCut were accepted for further use. Anipose is a 3D kinematic reconstruction package that utilizes triangulation and spatial and temporal filters to combine frame-based position data from multiple camera views to estimate absolute 3D position. The output of DeepLabCut was imported into Anipose with default settings, utilizing videos of a 10-by-7 square checkerboard (1 in square size) for calibration and triangulation. The output of this kinematic extraction pipeline was absolute 3D fingertip position in mm.

Fingertip position data were linearly interpolated over instances of dropped frames, and smoothed with a Gaussian kernel (50 ms standard deviation for peri-reach modulation analyses, 15 ms for regression analyses), and numerically differentiated to produce fingertip velocity. The vector magnitude of the 3D fingertip velocity was taken to produce scalar fingertip speed. Velocity was further differentiated to calculate 3D acceleration. Acceleration magnitude was calculated, as well as signed acceleration (the acceleration magnitude multiplied by the sign of the cross product of the velocity and acceleration vectors at each timepoint).

### Reach event detection and reach curation

To exclude irregular and non-task-related movements from analysis, videos were manually reviewed by an experimenter (R.N.T.) after sessions were complete. Trials in which subjects moved early or late, did not move, did not hold over targets, moved in the wrong direction, moved in multiple disjointed segments, or made otherwise aberrant movements were marked for exclusion. 14.7% (523/3553) of initial reaches were excluded in this way.

For valid trials, fingertip kinematics were utilized to determine the start and end times of each reaching movement. For reach detection, fingertip speed was first low pass filtered at 3 Hz in order to avoid the influence of tremor (characteristic ET tremor frequency 4–12 Hz). Then, for each trial, the time of peak speed in a window from 0.5 s before to 2.5 s after target display time was identified. Next, the average velocity in a 0.5 s window around peak speed time was calculated to determine the dominant reach direction vector. The reach-aligned velocity was then calculated by projecting 3D fingertip velocity from a window 2 s before to 2 s after peak speed time onto the dominant reach direction vector. Reach Start was defined as the last time before peak speed that the reach-aligned velocity became positive or displayed a minimum that was less than 5% of the peak reach-aligned velocity (to counteract effects of slow drifts or tremor in the reach direction). Reach End was defined as the first time after peak speed that the reach-aligned velocity became negative or displayed a minimum that was less than 5% of the peak reach-aligned velocity. Trials for which Reach Start or Reach End could not be identified using these criteria were excluded from further analysis. Reaches that were of duration longer than 1.5 times the interquartile range above the 3rd quartile of reach durations per hemisphere were discarded. Sessions for which fewer than 25 valid reaches could be identified were excluded from further analysis.

### Neural data acquisition and preprocessing

Neuronal spiking activity was recorded using Sonus shielded 3 mm micro tip platinum iridium NeuroProbe microelectrodes (AlphaOmega Engineering, Nof HaGalil, Isreal). Recordings were digitized and saved at 44 kHz using a Neuro Omega system (Alpha Omega Engineering). The center microelectrode recording trajectory was determined by routine combination of indirect and direct stereotactic targeting; consensus procedure was used to generate starting coordinates: 11 mm lateral to the wall of the third ventricle at a distance anterior to the posterior commissure equal to ¼ of the straight-line distance between the anterior commissure and the posterior commissure. Entry points were determined by clinicians to avoid vasculature and vital brain structures. Depending on clinician preference, two or three microelectrodes were inserted simultaneously for each procedure, utilizing the central trajectory aimed at the MRI-derived target and auxiliary parallel trajectories offset from the central trajectory by 2 mm in a posterior, anterior or medial direction in a Ben-Gun arrangement.

Neural signals were bandpass filtered between 300 and 3000 Hz, and threshold crossings were detected at −3 standard deviations to extract 800 ms long snippets. These snippets were then imported into Offline Sorter V3 (Plexon Inc, Dallas, TX) for manual spike sorting. Spiking units were sorted based on waveform shape, and clear discriminability from background activity that persisted throughout an entire task session. In cases where multiple spike sources could not be reliably separated, spikes were sorted into a “multi-unit.” Both single- and multi-units are referred to throughout the text as “units.” Recordings with no discriminable spikes were withheld from further analysis.

Spike times for each unit were utilized to calculate fractional interval FRs in 1 ms bins spanning entire task sessions. A Gaussian kernel (50 ms s.d. for peri-reach modulation analyses, 15 ms s.d. for regression analyses) was then convolved with these FRs to generate a smooth FR at desired timepoints around detected reach events. For regression analyses, FRs were further subjected to z-score normalization within a sliding 45 s window to counter the effects of drift in the recorded spike amplitude.

### Peri-reach firing rate modulation

To identify periods of significant FR modulation relative to reach events, FR was calculated for each unit in a time window spanning from 1.5 s before each Reach Start to 1.5 s after Reach End. For the pre- and post-reach windows, FR was calculated at 10 ms increments. Due to variable reach durations, and in order to directly compare across all reaches for all subjects, a time-stretching technique was used to calculate FRs between Reach Starts and Reach Ends. An equal number of evenly spaced samples (80) was used for each reach, resulting in variable resolutions on each reach, but an average resolution of 9.99 ms over all valid reaches by all subjects. This allowed for direct comparison of reaches regardless of duration. Analyses were also performed without this time-stretching procedure (Extended Data Fig. 1).

Each unit’s peri-reach FRs were then averaged at each timepoint across all reaches within a session to generate the mean peri-reach FR (Fig. 3a). To determine whether variation in the mean peri-reach FR qualified as significant modulation above or below the unit’s baseline activity, a Monte Carlo method was used to generate a shuffle-derived baseline distribution of mean FRs. For each unit, the following procedure was repeated 10,000 times: a sample, equal in size to the number of reaches completed in the task session, of single FR values was calculated at times chosen at random from the entire recording for that unit; the mean of this sample constituted a single entry in the shuffle-derived baseline mean FR distribution. Then, at each timepoint, the observed mean peri-reach FR was compared against the shuffle-derived baseline distribution to generate an empirical p-value (the proportion of baseline entries more extreme than the observed FR). To be considered significantly modulated, a unit was required to display significant empirical p-values (< 0.01, two-sided) for at least 50 ms (5 contiguous samples), and at least one of these modulation periods had to occur within a window spanning 400 ms before Reach Start to 400 ms after Reach End. Periods of modulation lasting less than 50 ms were rejected.

The grand mean fingertip speed profile (Fig. 3b) was calculated by re-sampling fingertip speed using cubic spline interpolation at the same peri-reach timepoints and averaging across all reaches across all subjects.

### Spatial activation heatmaps

Spatial activation heatmaps were calculated by computing the top 2 principal components (PCs) of 3D fingertip position, and dividing this top-two PC space into a grid of 15 mm squares. For each square, the FR was averaged across all kinematic timepoints when the finger fell within its boundaries (Fig. 4a).

### Directional tuning

Each recorded unit was assessed for reach direction sensitivity. Reaches for each task session were partitioned into groups according reach direction, regardless of whether the reach was outward or inward. For example, reaches from the central target to the upper right target (Target 4) were grouped together with reaches from the lower left target (Target 8) to the central target. This grouping was chosen to emphasize the true directional response by minimizing the influence of positional proprioception or muscle-related activity (i.e. different muscle and joint actions, but the same movement direction). The same peri-reach FR traces used in the peri-reach modulation analyses were used for the directional tuning analyses.

To determine if a unit exhibited significant directional tuning, at each peri-reach timepoint, FRs were subjected to Kruskal-Wallis ANOVA with FRs grouped according to reach direction. To qualify as a “significantly tuned” unit, the unit was required to display Kruskal-Wallis p-values < 0.05 for at least 50 ms (5 contiguous samples), with at least one of these modulation periods falling within the peri-reach window spanning 400 ms before Reach Start to 400 ms after Reach End.

### Regression analyses

The extent to which FRs encoded kinematics was investigated using multiple linear regression. For this analysis, FRs were calculated at the kinematic sample times, and smoothed with a 15 ms s.d. Gaussian kernel. Only kinematic timepoints occurring between 250 ms before each valid Reach Start and 250 ms after the subsequent Reach End were included.

For each unit, FRs at these reach-centered times were regressed against two sets of regressors: (1) a “kinematics only” set containing the fingertip’s 3D position, 3D velocity, speed, 3D acceleration, acceleration magnitude and signed acceleration; and (2) a “kinematics + error” set containing the “kinematics only” set of terms augmented by the inclusion of 3D error (the vector difference between instantaneous 3D position and the mean 3D position between 0 and 1 s after the next Reach End) and error magnitude (the magnitude of the 3D error vector). The time period of 0–1 s after Reach End equated to the early hold period, and the location was the cued target; taking the mean position largely eliminated variation due to tremor and small fluctuations of movement, resulting in a position that estimates the participant’s goal endpoint position. Regressions were repeated at a range of lags by shifting the timepoints at which the FRs were calculated in 8.33 ms increments (one kinematic sample time) up to 1 s before kinematic timepoints (negative lags) or 1 s after kinematic timepoints (positive lags).

All FRs and regressor terms were normalized by centering and dividing by their standard deviations, and multiple linear regression was used to generate R-square values for each unit at each lag for both regressor sets. Significance of these R-square values was assessed by comparing the observed R-square against a distribution of chance R-square values. Each entry in the chance R-square distribution was calculated by using FR timepoints that were time-shifted relative to the kinematic timepoints by a random amount that was allowed to span the entire length of the recording (a similar procedure to the lagged regressions but allowing the lag to span anywhere from 0 s to the length of the recording). In the case that the time-shift exceeded the length of the recording, FR values from the beginning of the register were appended to the end, using the MATLAB function *circshift*. This procedure was repeated to generate 10,000 chance R-squares, and to qualify as significant, an observed R-square was required to fall above the 99th percentile of the chance R-square distribution (α = 0.01, one-sided).

Additional regressions were performed using reach data from windows centered around either Reach Starts or Reach Ends. For these regressions, kinematic, error and neural data at zero lag were collected from 500 ms wide windows centered on the timepoints of interest. Significance of resulting R-squares was calculated using the random time-shifting method above.

The proportional contributions of each regressor to the overall R-square were evaluated using Shapley decomposition. Shapley decomposition is a method based on the Shapley Value^61^, that allows for the exact calculation of the portion of the full-model R-square that can be assigned to each individual regressor in a multiple linear regression model in a way that is robust to multicollinearity in the regressors^62^. This method extends the concept of the marginal contribution of a regressor *x*_*i*_, which is the increase in R-square observed when regression is performed with a set of regressors including *x*_*i*_ compared to regression performed with the same set of regressors, excluding *x*_*i*_. Under Shapley decomposition, the total contribution of regressor *x*_*i*_ to the full-model R-square is found by calculating a weighted average of the marginal contribution of *x*_*i*_ to all possible reduced models (that is, models containing every possible combination of other regressors). This method was repeated for all regressors in a model, generating absolute contributions for each regressor that sum to the full-model R-square^62^.

Shapley decomposition was performed for each significantly encoding unit to calculate the absolute contribution of each individual regressor to full-model R-square. Dividing absolute contribution by the full-model R-square yields proportional contribution. For the 3D terms (e.g. 3D position, 3D error), the contributions from x-, y- and z-components were summed to generate a total contribution for that 3D feature group. Contributions for scalar terms (e.g. speed, error magnitude) were taken as is.

## Data Availability

The data that support the findings of this study are available in the following Open Science Framework repository: https://osf.io/3unka/?view_only=4e7e0d5cee0e4dd9a567fa637644b3b9
